# Supplementary material for: A comprehensive stroke risk assessment by combining atrial computational fluid dynamics simulations and functional patient data
Source: Sci Rep. 2024 Apr 25;14:9515. doi: 10.1038/s41598-024-59997-2 (PMC11045804; doi:10.1038/s41598-024-59997-2)
Supplement: Supplementary file 1 — Supplementary Information. [file 41598_2024_59997_MOESM1_ESM.pdf]

A comprehensive stroke risk assessment by combining atrial computational fluid dynamics simulations and functional patient data
Supplementary Information

A. Zingaro, Z. Ahmad, E. Kholmovski, K. Sakata, L. Dede', A. K. Morris, A. Quarteroni, N. A. Trayanova

1 Supplementary Results

We report supplementary results from the study. In Supplementary Table 1, we show medians of the boxplot of hemodynamic features computed with our patient-specific CFD simulations in the LA and LAA. In Supplementary Figure 1 we display the boxplot of the hemodynamics features for each patient, focusing on the LAA only (result on the LA are given in the paper). Supplementary Figure 2 shows the LA vorticity magnitude in three different time instants plot against all the functional data considered. Similarly, in Supplementary Figure 3 we show the same results but focused on the LAA (plotted against all the LAA functional data considered).

			**Control**				**Stroke**			
			**C1**	**C2**	**C3**	**C4**	**S1**	**S2**	**S3**	**S4**
**LA**	FS	[–]	0.3019	0.3500	0.1487	0.2960	0.4875	0.3962	0.5254	0.3262
	TAWSS	[Pa]	0.6874	0.8157	0.8906	0.8316	0.5922	0.5556	0.5916	0.8205
	OSI	[–]	0.0804	0.0868	0.0742	0.0636	0.0861	0.1278	0.1371	0.0757
	RRT	[1/Pa]	1.8647	1.6056	1.4122	1.4223	2.3328	2.6041	2.6568	1.4575
	ECAP	[1/Pa]	0.1307	0.1048	0.0947	0.0764	0.1673	0.2433	0.2586	0.0899
	$\|\boldsymbol{\omega}\|^{(E)}$	[1/s]	153.2700	131.4600	141.3100	109.1700	136.0800	118.0900	105.8000	154.3700
	$\|\boldsymbol{\omega}\|^{(A)}$	[1/s]	107.3800	127.7200	108.0900	124.3200	124.6700	115.5500	133.2500	135.3000
	$\|\boldsymbol{\omega}\|^{(S)}$	[1/s]	26.2120	23.1450	43.6410	28.5460	18.0620	19.7810	14.3170	30.6820
**LAA**	FS	[–]	0.3019	0.3500	1.0000	0.8305	0.4875	0.3962	0.7797	0.3262
	TAWSS	[Pa]	0.6874	0.8157	0.0621	0.1110	0.5922	0.5556	0.1770	0.8205
	OSI	[–]	0.0804	0.0868	0.2130	0.0457	0.0861	0.1278	0.0999	0.0757
	RRT	[1/Pa]	1.8647	1.6056	28.5008	11.4786	2.3328	2.6041	8.1796	1.4575
	ECAP	[1/Pa]	0.1307	0.1048	2.9830	0.4140	0.1673	0.2433	0.6936	0.0899
	$\|\boldsymbol{\omega}\|^{(E)}$	[1/s]	12.7880	16.9670	8.7049	11.2150	6.5866	20.7240	6.8452	6.1957
	$\|\boldsymbol{\omega}\|^{(E)}$	[1/s]	15.0590	110.7100	10.6680	44.7410	14.7020	85.4180	40.5560	9.5050
	$\|\boldsymbol{\omega}\|^{(E)}$	[1/s]	14.9870	25.4920	16.2610	11.3740	12.2680	11.5500	10.1500	13.9110

Supplementary Table 1. Medians of boxplot of hemodynamic features computed with patient-specific CFD simulations in the LA and LAA for control and stroke cases.

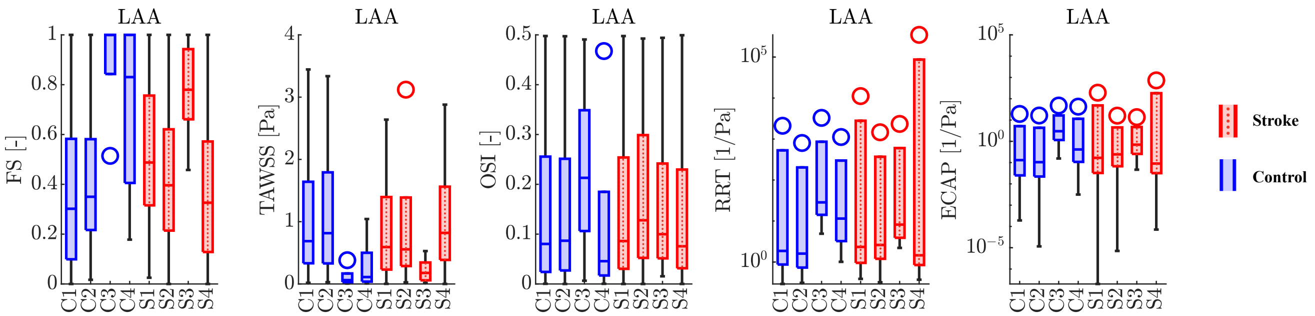

Supplementary Figure 1. Boxplots of hemodynamics features computed with patient-specific CFD simulations in the LAA. From the left to the right: FS, TAWSS, OSI, RRT, and ECAP. For each indicator, the first four boxplots are control cases, the last four are stroke cases.

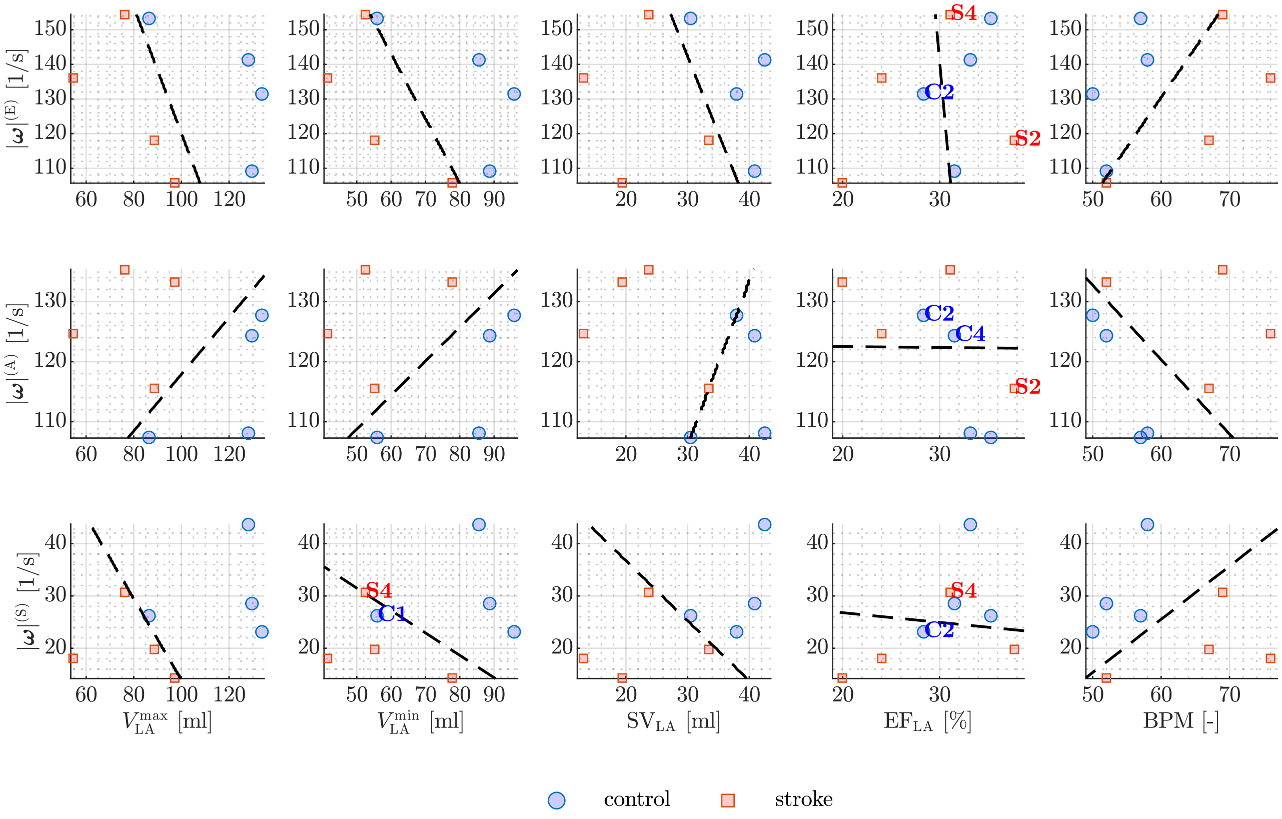

Supplementary Figure 2. Medians of vorticity magnitude in the LA against functional data from CMR. The black-dashed line is obtained by running logistic regression. From top to the bottom: vorticity magnitude at E-wave, A-wave, and systolic peak (MV closed) are plotted against maximum LA volume, minimum LA volume, LA SV, LA EF, and BPM (from left to right). Outliers are denoted in each plot (when present).

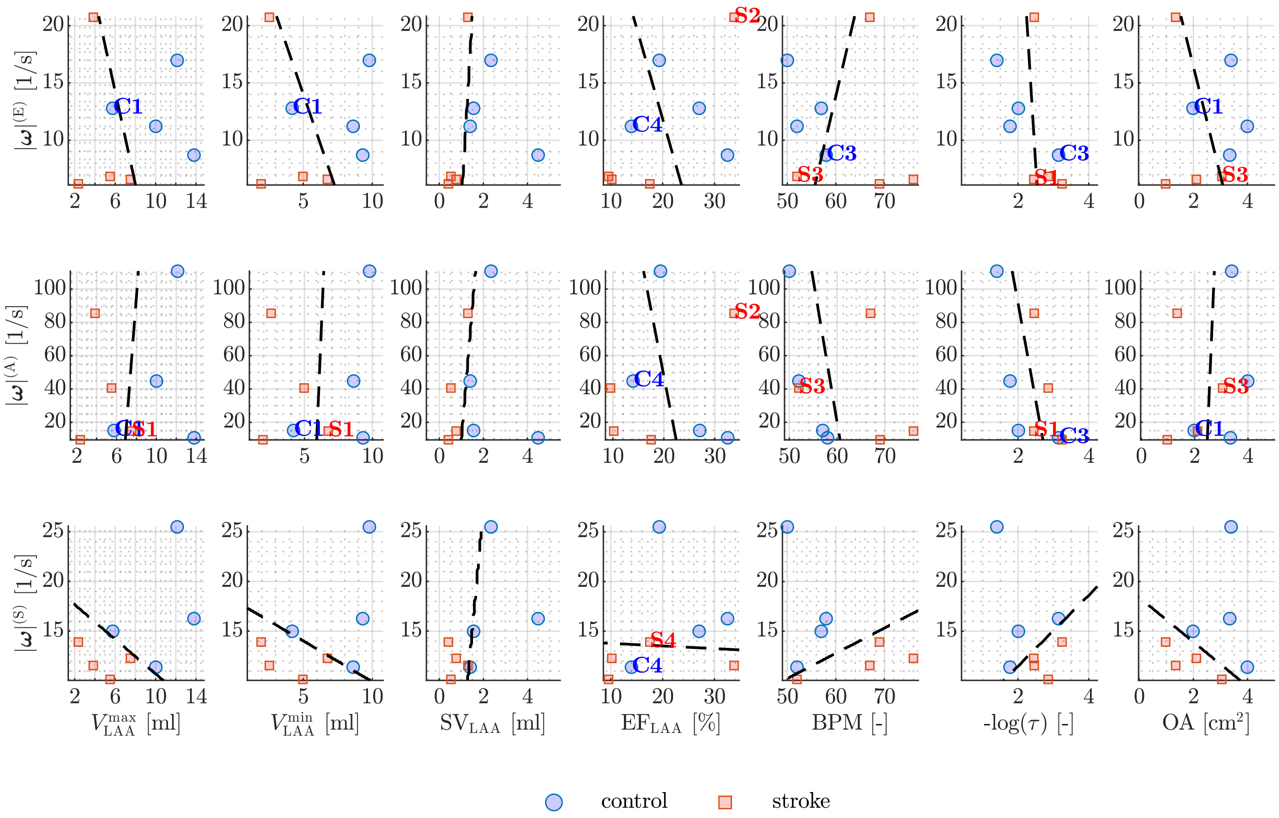

Supplementary Figure 3. Medians of vorticity magnitude in the LAA against functional data from CMR. The black-dashed line is obtained by running logistic regression. From top to the bottom: vorticity magnitude at E-wave, A-wave, and systolic peak (MV closed) are plotted against maximum LAA volume, minimum LAA volume, LAA SV, LAA EF, BPM, tortuosity, and OA (from left to right).Outliers are denoted in each plot (when present).

2 Smoothing spline approximation of the MRI displacement

The patient-specific displacement field from MRI $\boldsymbol{y} = [y^{(1)}, y^{(2)}, y^{(3)}]^T$ is defined for a discrete set of points n: $(t_i, \boldsymbol{y}_i)$ with $i = 0, \dots, n$. Let $y^{(k)}$ be the displacement in a generic direction, with $k = 1,2,3$. For each direction k, we define the smoothing spline $\widetilde{f}^{(k)} : [0, T_{\mathrm{HB}}] \to \mathbb{R}$ as that function that minimizes the following functional:

$$J^{(k)}(f) = \sum_{i=0}^{n} \left(f(t_i) - y_i^{(k)} \right)^2 + \lambda \int_0^{T_{\mathrm{HB}}} f''(t)\,\mathrm{d}t,$$

where $\lambda > 0$ is a regularization weight. Notice that, the case $\lambda = 0$ corresponds to interpolant cubic spline, whereas for $\lambda \to \infty$, the approximant tends to the least-squares regression line of the input points. In this paper, we set $\lambda = 0.2$. We discretize the CFD temporal domain as $t_j = j\Delta t$, for $j = 1, \dots, N_t$, with Δt the time step size (much smaller than the one of MRI data). The displacement for the simulation will consist of the evaluation of $\widetilde{f}^{(k)}(t)$ in the discretized time t_j, for $j = 1, \dots, N_t$ and $k = 1,2,3$.
